# SYTL2 promotes metastasis of prostate cancer cells by enhancing FSCN1-mediated pseudopodia formation and invasion

**DOI:** 10.1186/s12967-023-04146-y

**Published:** 2023-05-05

**Authors:** Zean Li, Yiran Tao, Ze Gao, Shirong Peng, Yiming Lai, Kaiwen Li, Xu Chen, Hai Huang

**Affiliations:** 1 Department of Urology, Sun Yat-Sen Memorial Hospital, Sun Yat-Sen University, Guangzhou, 510120 China; 2 Guangdong Provincial Key Laboratory of Malignant Tumor Epigenetics and Gene Regulation, Sun Yat-Sen Memorial Hospital, Sun Yat-Sen University, 107. W. Yanjiang Road, Guangzhou, 510120 China; 3 Guangdong Provincial Clinical Research Center for Urological Diseases, Sun Yat-Sen Memorial Hospital, Sun Yat-Sen University, Guangzhou, 510120 China; 4 Department of Urology, The Sixth Affiliated Hospital of Guangzhou Medical University, Qingyuan People’s Hospital, Qingyuan, 511518 Guangdong China; 5 Medical Research Center, Sun Yat-Sen Memorial Hospital, Sun Yat-Sen University, Guangzhou, 510120 China; 6 Department of Urology, The Six Affiliated Hospital, Sun Yat-Sen University, Guangzhou, 510655 China; 7 Department of Urology, Qilu Hospital of Shandong University, Jinan, 250000 China

**Keywords:** SYTL2, Pseudopodia, Prostate cancer, Metastasis, FSCN1

## Abstract

**Background:**

Metastatic prostate cancer (mPCa) has a poor prognosis with limited treatment options. The high mobility of tumor cells is the key driving characteristic of metastasis. However, the mechanism is complex and far from clarified in PCa. Therefore, it is essential to explore the mechanism of metastasis and discover an intrinsic biomarker for mPCa.

**Methods:**

Transcriptome sequencing data and clinicopathologic features of PCa from multifarious public databases were used to identify novel metastatic genes in PCa. The PCa tissue cohort containing 102 formalin-fixed paraffin-embedded (FFPE) samples was used to evaluate the clinicopathologic features of synaptotagmin-like 2 (SYTL2) in PCa. The function of SYTL2 was investigated by migration and invasion assays and a 3D migration model in vitro and a popliteal lymph node metastasis model in vivo*.* We performed coimmunoprecipitation and protein stability assays to clarify the mechanism of SYTL2.

**Results:**

We discovered a pseudopodia regulator, SYTL2, which correlated with a higher Gleason score, worse prognosis and higher risk of metastasis. Functional experiments revealed that SYTL2 promoted migration, invasion and lymph node metastasis by increasing pseudopodia formation in vitro and in vivo. Furthermore, SYTL2 induced pseudopodia formation by enhancing the stability of fascin actin-bundling protein 1 (FSCN1) by binding and inhibiting the proteasome degradation pathway. Targeting FSCN1 enabled rescue and reversal of the oncogenic effect of SYTL2.

**Conclusions:**

Overall, our study established an FSCN1-dependent mechanism by which SYTL2 regulates the mobility of PCa cells. We also found that the SYTL2-FSCN1-pseudopodia axis may serve as a pharmacological and novel target for treating mPCa.

**Supplementary Information:**

The online version contains supplementary material available at 10.1186/s12967-023-04146-y.

## Introduction

Metastatic prostate cancer exhibits unique characteristics compared with localized PCa. When malignant cells escape from primary PCa, settle into distant organs and eventually form clinically detectable metastatic lesions, the five-year survival rate decreases to 29% [1–3]. Metastasis is a multistep process regulated by various factors involved in tumor-intrinsic mechanisms, including oncogenic mutation, phenotypic switching and cytoskeleton reconstruction, and tumor-extrinsic mechanisms, such as extracellular matrix (ECM) remodeling, inflammatory microenvironment generation and angiogenesis [4–6]. The regulators of metastasis vary, and the mechanism of metastatic PCa (mPCa) remains unclear. Therefore, it is essential to explore the mechanism of PCa metastasis and discover an intrinsic biomarker for PCa.

Intrinsic tumor changes are the first and decisive step in PCa metastasis. For example, cytoskeleton reconstruction and pseudopodia formation govern the migration rate of malignant prostate cells [7–10]. The formation of pseudopodia, which depends on actin dynamics, is critical for altering the morphology of tumor cells during metastasis [11, 12]. Currently, the literature has noted that several signaling axes, such as DOCK7/RAGE/CDC42 and Hop/RhoC, are involved in pseudopodia formation [13]. Additionally, dishevelled associated activator of morphogenesis 1 (Daam1) regulates the formation of pseudopodia by interacting with fascin actin-bundling protein 1 (FSCN1), a key factor in the organization of actin filament bundles and the formation of membrane ruffles, via the FH2 domain [14–16]. Understanding the mechanisms underlying pseudopodia formation in PCa will be instrumental in addressing the problem of mPCa.

Synaptotagmin-like 2 (SYTL2), also known as SLP2, is located on chromosome 11q14.1 [17]. SYTL2 is a member of the C2 domain-containing protein family, which is the regulator of vesicular trafficking involved in the Rab family and cytoskeleton reconstruction [18]. It contains an N-terminal Slp homology domain (SHD) and interacts with the Ras-related protein RAB-27A for vesicular trafficking and remodeling the size of renal cells [19–22]. Several studies have demonstrated that SYTL2 expression is positively correlated with a poor prognosis in ovarian cancer, bladder cancer and gastric cancer and an advanced metastatic capacity [23–25]. However, the detailed biological function and mechanism of SYTL2 in PCa remain largely unknown.

In this study, the expression level of SYTL2 was found to be positively correlated with the Gleason score, advanced metastasis status and poor prognosis in mPCa patients. SYTL2 also interacts with FSCN1 and inhibits its degradation, enhancing the formation of pseudopodia and metastasis in PCa.

## Methods and materials

### Data analysis of databases

Two datasets (GSE45016 [26] and GSE67872 [27]) from the Gene Expression Omnibus (https://www.ncbi.nlm.nih.gov/geo/) were analyzed by the GEO2R tool. The GSE45016 dataset (including three nonmetastatic samples and seven mPCa samples) and GSE67872 (including three normal samples, four localized PCa samples and three mPCa samples from prostate-specific loss of Pten and K-ras activation mice) were based on the GPL570 and GPL10361 platforms, respectively.

The transcriptome sequencing data of SYLT2 and the clinical information of the Chinese Prostate Cancer Genome and Epigenome Atlas (CPGEA), including 208 pairs of primary PCa and matched tumor-adjacent samples from Chinese patients, 136 pairsof which had the transcriptome sequencing data.  [28], and The Cancer Genome Atlas (TCGA), including 489 PCa samples and 51 tumor-adjacent prostate tissue samples, were collected to perform data analysis and disease-free survival analysis, respectively. Cases without survival data or transcriptome sequencing data were excluded from the analysis. The PCa samples from TCGA and CPGEA were classified as having low or high levels of SYTL2 expression by using the median.

### Human tissue samples

A total of 102 paraffin-embedded PCa tissues (FFPE-SYSCC cohort), including 66 radical prostatectomy (RP) specimens and 36 transurethral resection prostate (TURP) specimens, were obtained at Sun Yat-sen University Cancer Center (Guangzhou, China) between January 2000 and August 2018. All patients provided written informed consent for the use of their tissues. The TURP samples were collected before 2012, and the patients did not tolerate radical prostatectomy, so palliative TURP surgery was performed to relieve their symptoms. All samples were diagnosed with PCa by two independent pathologists. The TNM stage and Gleason score of the PCa samples were confirmed according to the guidelines. Ethical approval was obtained from Sun Yat-sen University’s Committees for Ethical Review of Research Involving Human Subjects. All patients were followed up until December 2018.

### Immunohistochemical (IHC) staining and scoring analyses

IHC staining was performed as previously described [29]. Briefly, paraffin sections of the PCa samples were deparaffinized and hydrated. The microwave method and 0.3% H_2_O_2_ were used to retrieve the antigen and block endogenous peroxidase activity, respectively. For immunohistochemical staining, primary antibodies against SYTL2 (1:100, PA5-24730, Thermo, USA) were added, followed by secondary antibodies and DAB color development. The sections were counterstained with hematoxylin and mounted in non‐aqueous mounting medium.

The expression of SYTL2 in the PCa tissues was blindly quantified by two pathologists. The score was then calculated as the intensity score (negative = 0, weak = 1, moderate = 2, or strong = 3) multiplied by the proportion score (positively stained cells < 25% = 1, 25–50% = 2, 50–75% = 3 and > 75% = 4) (score = intensity × proportion score). The samples were classified as having low (score ≤ 6) or high (score > 6) SYTL2 expression. Images were obtained by a Nikon Eclipse Ni-U (Nikon, Japan) microscope system and quantified with NIS-Elements software.

### Cell culture

Human PCa cell lines (DU145 and PC3) were provided by the Cell Bank of the Chinese Academy of Sciences (Shanghai, China), and human embryonic kidney-293 (HEK-293) cells were purchased from ATCC (American Type Culture Collection. Manassas, VA, USA). DU145 and 293T cells were cultured in DMEM (Gibco, Shanghai, China), whereas PC3 cells were cultured in RPMI 1640 (Gibco, Shanghai, China) with 10% FBS and 1% penicillin/streptomycin (Gibco, Shanghai, China). Cycloheximide (CHX, used at 50 µg/ml, M4879) and MG132 (10 μM, M1902) were purchased from Abmole (Houston, TX, USA). The cells were cultured in a humidified atmosphere of 5% CO_2_ at 37 °C (BB150, Thermo Scientific, Shanghai, China).

### RNA isolation and qRT‒PCR

Total RNA was isolated using RNAiso Plus (9109, TaKaRa, Japan) according to the manufacturer’s instructions. RNA was reverse transcribed into cDNA using the PrimeScript RT Reagent Kit (RR047A, TaKaRa, China). The expression of mRNA in prostate cell lines was quantified by an ABI QuantStudio Sequence Detection System (Applied Biosystems). Each reaction was performed in triplicate. The specific primers purchased from IGEbio are listed in Additional file 1: Table S1.

### Transient transfection

RNA interference (siRNA) oligonucleotides targeting SYTL2 and FSCN1 and negative control siRNAs were purchased from GenePharma (Shanghai, China). The siRNA sequences are listed in Additional file 1: Table S1. Then, 100 nM siRNA with 3 μl/ml Lipofectamine RNAimax (Life Technologies, Waltham, MA, USA) was added to the cell culture and incubated for 48 h for RNA isolation and 72 h for protein collection.pcDNA3.1 FSCN1 (AGEbio, Guangzhou, China) or an empty vector was transiently transfected into prostate cells with X-tremeGENE HP DNA Transfection Reagent (6366546001, Roche, Basel, Switzerland), cultured for 48 h, and then subjected to further investigation.

### Western blotting

Western blotting was performed as previously described[30]. Primary antibodies specific to SYTL1 (1:500, PA5-24730, Thermo), FSCN1 (1:1000, ab126772, Abcam), ubiquitin (1:500, 10201, Proteintech, Wuhan, China), and GAPDH (1:1000, AA128, Beyotime, Shanghai, China) were used. The secondary antibodies were purchased from Cwbiotech (1:10,000, Beijing, China), and the blots were visualized using Immobilon Western Chemiluminescent HRP Substrate (WBKLS0500, Merck Millipore, Germany).

### Coimmunoprecipitation (Co-IP) assays

Co-IP assays of SYTL1 and FSCN1 were performed as described in our previous study[31] with the Pierce Crosslink Magnetic IP/Co-IP Kit (88805, Thermo Scientific, Shanghai, China). Briefly, 5 μg of an anti-Flag (14793), anti-IgG (3900, CST) or anti-FSCN1 (1:1000, ab126772, Abcam) antibody was bound to Protein A/G Magnetic Beads for 1 h at room temperature. The antibody-crosslinked beads were then incubated overnight at 4 °C with 400 μg of cell lysate that contained Flag-SYTL2 and FSCN1. The proteins interacting with the antibodies were eluted from the magnetic beads. The samples were then used for SDS‒PAGE immunoblot analysis, and the SDS‒PAGE gel was silver-stained by using the Pierce Silver Stain Kit (24612, Thermo Scientific) or subjected to mass spectrometry (MS) analysis with an Easy nanoLC 1200—Orbitrap Fusion (Thermo Fisher, USA).

### Lentivirus transduction

To establish stable knockdown and overexpression, cell lines were constructed as described previously [29, 32]. Briefly, full-length SYTL2 or shRNA sequences targeted to SYTL2 were cloned into the vectors pCDH-GFP-CMV-EF1-Puro-3xFlag or pLKO.1-Puro. The sequences of the shRNAs are listed in Additional file 1: Table S1. Lentivirus production and infection were conducted as described previously.

### Protein stability assays

The PCa cell line PC3, with or without stable knockdown of SYTL2, was plated at 5 × 10^5^ cells in six-well plates. Following adhesion, the cells were treated with cycloheximide (CHX, 50 µg/ml) for 2 h, 4 h, or 8 h to inhibit protein synthesis. The cell lysates were prepared and analyzed by western blotting. FSCN1 protein bands were quantified using ImageJ. GAPDH was used as the loading control. The degradation rate of FSCN1 was displayed as the half-life (t1⁄2), which is the time required for 50% of the protein to be degraded.

The proteasome inhibitor MG-132 (10 µM) was added to the PC3 cell line in six-well plates to inhibit protein degradation. Cell lysates were prepared as described above. The protein abundance was analyzed by western blotting.

### Ubiquitination assays

PC3 cell lines, with or without stable overexpression of SYTL2, were seeded in 150 mm^2^ plates. Following adhesion, the cells were treated with MG-132 (10 µM), and cell lysates were harvested and incubated with anti-FSCN1 (ab126772, Abcam) as described for the Co-IP assay. The cell lysates eluted from the magnetic beads were immunoblotted with the anti-Ub antibody (1:500, 10201, Proteintech).

### Cell migration and invasion assays

Cell migration and invasion assays were performed as previously described [33]. Briefly, 40,000 cells/well in serum-free DMEM or RPMI 1640 were seeded into the top culture insert with 8-μm pores (353097, Corning, USA), and the bottom well was filled with DMEM or RPMI 1640 medium containing 10% FBS. Following incubation for 24 h at 37 °C, the membranes were stained using crystal violet. The cells in the bottom chamber were counted using an Olympus IX71 inverted microscope (Olympus, Japan). The PET membranes were covered with Matrigel Basement Membrane Matrix (354234, Corning, NY, USA) for the cell invasion assays.

### 3D cell migration model

The 3D cell migration model was established as previously described [29]. One hundred microliters of Matrigel (354234, Corning, NY, USA) was placed in a 24-well plate, and the cell suspension (3000/well) was mixed with the gel (vol:vol = 1:1) and then incubated for 1 h at 37 °C to allow the gel to solidify. The culture medium was added to the plate after 24 h, and the plate was then cultured for 72 h followed by observation with a Nikon Eclipse Ni-U upright microscope (Nikon, Tokyo, Japan).

### Actin cytoskeleton staining

Actin cytoskeleton staining was performed to observe the formation of pseudopodia. The PCa cell line was seeded in confocal dishes at 1 × 10^5^ cells/well. Confocal dishes were washed three times with PBS followed by fixation with 4% paraformaldehyde (PFA, YJ0002, YongJing bio, Guangzhou, China) for 15 min. After another three washes with PBS, rhodamine phalloidin (1:100, RM02835, Abclonal, Wuhan, China) was added to the dishes and incubated for 1 h at 37 °C in the dark. The nuclei were stained with DAPI (2 μg/ml, G1012, Servicebio, Wuhan, China), and all incubations were followed by 3 PBST washes. Before collecting the images under a confocal microscope (Zeiss LSM 800), the cells were sealed with anti-fluorescence attenuation sealant (S2100, Solarbio, Beijing, China).

### In vivo metastasis and tumorigenesis experiments

The in vivo metastasis assay was performed as previously described [29]. The Institute Animal Care and Use Committee of Sun Yat-sen University approved all procedures involving animals. Male BALB/c nude mice (4–6 weeks old) were purchased from the Experimental Animal Center of Sun Yat-sen University and housed in specific pathogen-free (SPF) barrier facilities. PC3 cells (5 × 10^6^ cells) stably expressing firefly luciferase, which can be captured by a bioluminescence imaging system (Cypris FIS-250D (Xupu, China)), were inoculated into the mouse footpads. Eight weeks after the injections, the mice were euthanized, and the popliteal lymph nodes (LNs) were embedded in paraffin and analyzed using hematoxylin–eosin (H&E) staining. Images were obtained using a Nikon Eclipse Ni-U system with NIS-Elements software (Nikon, Tokyo, Japan). The volumes of the lymph nodes were calculated using the following formula: Lymph node volume (mm^3^) = (length [mm]) × (width [mm])^2^ × 0.5. The weights of the lymph nodes were also recorded.

### Statistical analyses

The quantitative data obtained from three independent experiments are presented as the means ± SDs. To compare the differences between two groups, an unpaired t test (two-tailed) was performed. One-way ANOVA followed by Dunnett’s multiple comparison test was performed to compare more than two groups. Factorial design was performed to determine the relationship between SYTL2 and FSCN1, and it was analyzed by two-way ANOVA.

Pearson’s χ^2^ test was used to analyze the clinical variables. Survival analysis was performed using the Kaplan‒Meier method and log-rank test. To statistically analyze the data, SPSS 20.0 software (SPSS, Armonk, NY, USA) or GraphPad Prism 5.0 (GraphPad, La Jolla, CA, USA) was used. A p value < 0.05 was considered significant.

## Results

### SYTL2 is an oncogenic gene for mPCa and correlates with metastasis and a poor prognosis

**Fig. 1:**
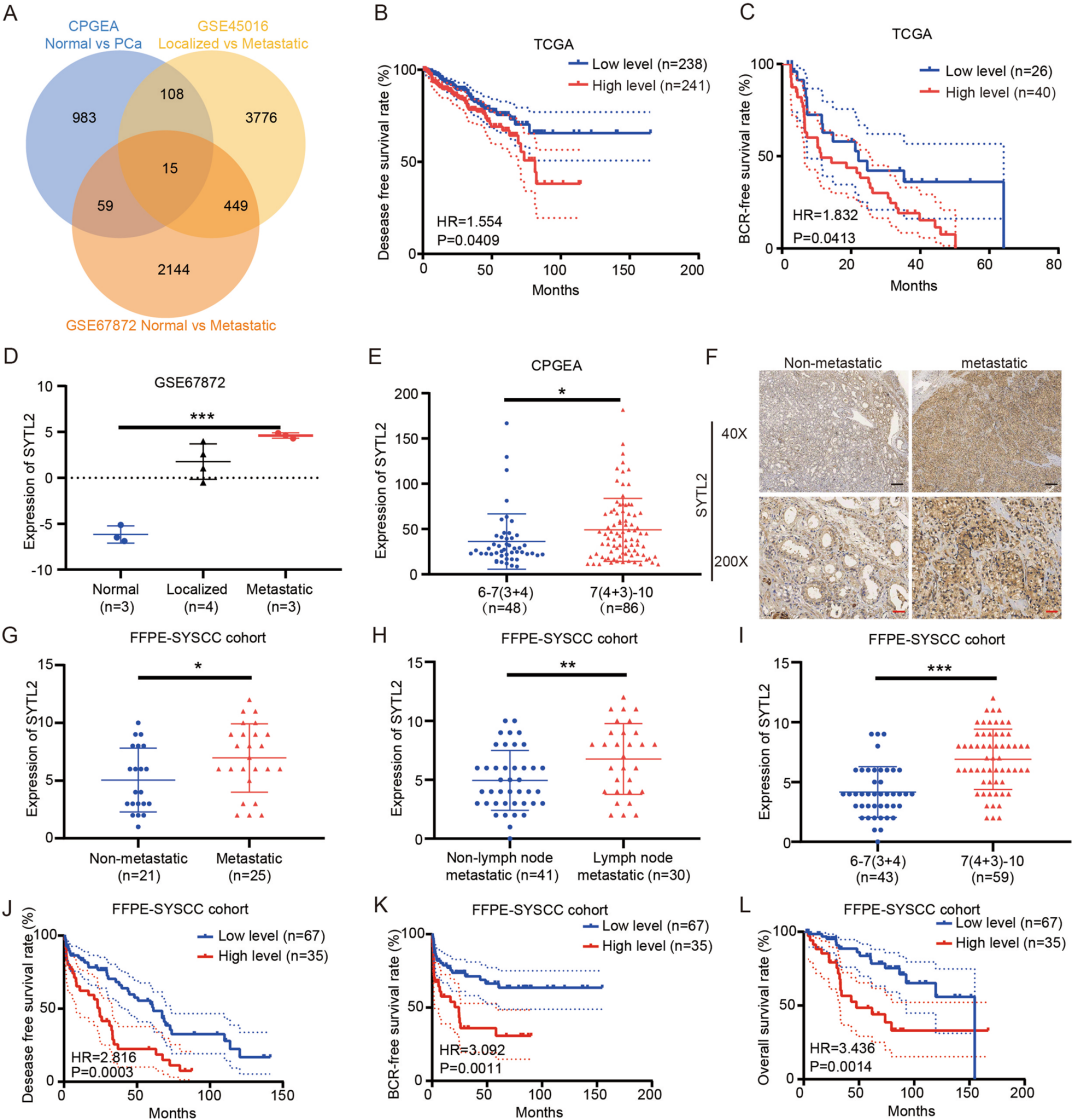
The expression level of SYTL2 is correlated with metastasis and poor prognosis in PCa patients. **A** Venn diagram shows the intersecting genes with high expression in PCa among the CPGEA database (primary PCa vs adjacent nontumor samples, logFC > 1.2, p < 0.05) and two GEO datasets, GSE45016 (mPCa vs localized PCa) and GSE67872 (mPCa vs normal, logFC > 1.2, p < 0.05). **B**, **C** Kaplan‒Meier curves for DFS (**B**) and BCR-free survival (**C**) of PCa patients with high versus low expression of SYTL2 in TCGA. **D** The expression of SYTL2 in GSE67872 compared among normal, localized PCa and mPCa samples. **E** The expression of SYTL2 in CPGEA that compared Gleason scores 6–7 (3 + 4) with Gleason scores 7 (4 + 3) − 10. **F** Representative images of SYTL2 expression in mPCa tissues and localized PCa tissues. **G**–**I** The expression of SYTL2 in the FFPE-SYSCC cohort that compared nonmPCa with mPCa (**G**), nonlymph node metastatic tissue with lymph node metastatic tissue (**H**) and Gleason scores 6–7 (3 + 4) with Gleason scores 7 (4 + 3) − 10 (**I**). **J**–**L** Kaplan‒Meier curves for DFS (**J**), BCR-free survival (**K**) and OS (**L**) of PCa patients with high versus low expression of SYTL2 in the FFPE-SYSCC cohort. *p < 0.05, **p < 0.01 and ***p < 0.001, ns no significant, Scale bars: 250 μm (black); 50 μm (red)

To identify potential oncogenes in PCa, a comprehensive screening was performed with the data from CPGEA, TCGA and two datasets in the GEO database (Fig. 1A). First, we compared the expression level between primary PCa and matched tumor-adjacent samples of CPGEA and identified 1165 genes that were expressed at higher levels in PCa tissue (log FC > 1.2). Next, 4348 genes and 2667 genes that were expressed at much higher levels in mPCa samples were identified from the GSE45016 and GSE67872 datasets, respectively (log FC > 1.2). We found 15 genes that were expressed at higher levels in mPCa in an intersection among CPGEA and the two GEO datasets (Fig. 1A; Additional file 1: Table S2). However, we further determined that only one gene, SYTL2, was negatively correlated with both disease-free survival (DFS) and biochemical recurrence (BCR)-free survival in TCGA (Fig. 1B, C). Moreover, SYTL2 expression was much higher in mPCa samples than in localized and tumor-adjacent samples (Fig. 1D). Meanwhile, the expression level of SYTL2 was positively correlated with the Gleason score in CPGEA (Fig. 1E; Additional file 1: Table S3). These results demonstrated that SYTL2 positively correlated with malignancy and metastatic status.

To more deeply determine the specific relationship between SYTL2 and the clinicopathologic features of PCa, the protein level of SYTL2 was analyzed in a sample cohort containing 102 PCa FFPE samples. We found that the protein level of SYTL2 was significantly higher in metastatic PCa (mPCa) than in nonmetastatic tumors (P = 0.0297, Fig. 1F, G). Moreover, the expression of SYTL2 was positively correlated with a higher incidence of LN metastasis (P = 0.0075, Fig. 1H) and a higher Gleason score (P < 0.0001, Fig. 1I and Table 1). A positive relationship between SYTL2 and Gleason score was also found in the TCGA and CPGEA databases (Additional file 1: Tables S3, S4). In addition, SYTL2 was related to poor T stage in TCGA (Additional file 1: Table S4) and higher PSA levels in CPGEA (Additional file 1: Table S4). Furthermore, Kaplan‒Meier survival analysis of our data demonstrated that high expression of SYTL2 was associated with shorter overall survival (OS), BCR-free survival and DFS (Fig. 1J–L). All of these results demonstrated that SYTL2 was associated with the metastasis and poor prognosis of PCa.

**Table 1:** Associations between SYTL2 expression and clinicopathological characteristics of PCa patients in the FFPE-SYSCC cohort

Clinical feature	Total patients n	Low n	High n	P value
Age				
≤ 65	45	29	16	0.814
> 65	57	38	19	
Gleason score				
≤ 3 + 4	43	39	4	< 0.001^***^
≥ 4 + 3	59	28	31	
T state				
T1–2	20	15	5	0.237
T3–4	50	30	20	
Lymph node metastasis				
N0	41	32	9	0.006^**^
N1	30	14	16	
Distant metastasis				
M0	21	15	6	0.108
M1	25	12	13	

A total of 102 paraffin-embedded PCa tissues were collected. Some samples lacked clinicopathological information, in which 32 samples lacked T stage information and 31 lacked lymph node metastasis information. Only 46 samples obtained distant metastasis information. Bold indicates p-value < 0.05 was considered significant. **p < 0.01, ***p < 0.001

### SYTL2 promotes the migration and invasion of PCa cells in vitro

**Fig. 2:**
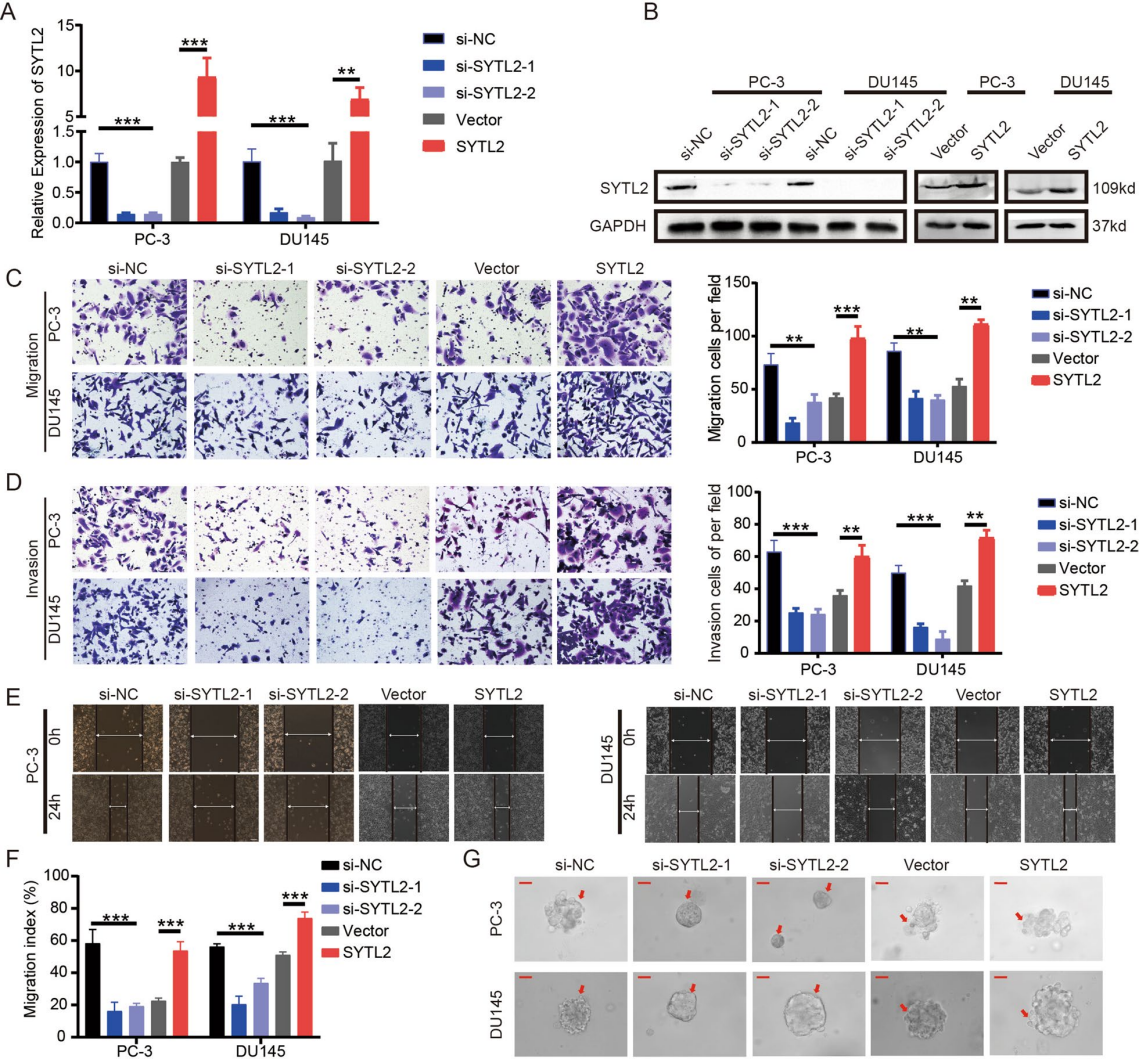
SYTL2 promotes the mobility of PCa cells in vitro. **A**, **B** qRT‒PCR (**A**) and Western blot (**B**) analysis of SYTL2 expression levels in SYTL2 knockdown, overexpressing cells and control cells. **C**, **D** Representative images of migration (**C**) and invasion (**D**) assays using PC-3 and DU145 cells (left panels) and a quantification analysis of migrated or invaded cell counts (right panels), showing cell migration and invasion after downregulation or upregulation of SYTL2. **E** Representative images of wound-healing assays using PC-3 and DU145 cells showing cell motility after downregulation or upregulation of SYTL2. **F** Quantification analysis of the cell migration index is shown. **G** Representative images of three-dimensional (3D) cell culture using PC-3 and DU145 cells, showing the cell invasive ability in stereoscopic space. *p < 0.05, **p < 0.01 and ***p < 0.001. Scale bars: 25 μm (red)

To examine the function of SYTL2 in PCa, two small interfering RNAs (siRNAs) targeting SYTL2 and lentivirus overexpressing SYTL2 were transfected into DU145 and PC3 cell lines. The results showed that the mRNA and protein levels of SYTL2 were significantly downregulated or increased in the DU145 and PC3 cell lines, respectively (Fig. 2A, B). Then, metastasis-related experiments, including cell migration, invasion, wound-healing assays and three-dimensional (3D) cell culture, were performed to explore the role of SYTL2 in regulating the motility capacity of PCa cells.

Cell migration and invasion assays revealed that a lower expression level of SYTL2 resulted in fewer migrating and invading cells, while a high level of SYTL2 showed a stronger metastatic capability (Fig. 2C, D). Wound-healing assays indicated the same results as the migration and invasion assays (Fig. 2E, F). Moreover, the 3D culture models, which mimicked the extracellular matrix environment, demonstrated that SYTL2-knockdown cells had an inhibited invasive capability, while overexpressing SYTL2 accelerated the invasion of PCa cells (Fig. 2G). Taken together, these data demonstrated that SYTL2 played a crucial role in PCa migration and invasion.

### SYTL2 facilitates lymph node metastasis of PCa cells in vivo

**Fig. 3:**
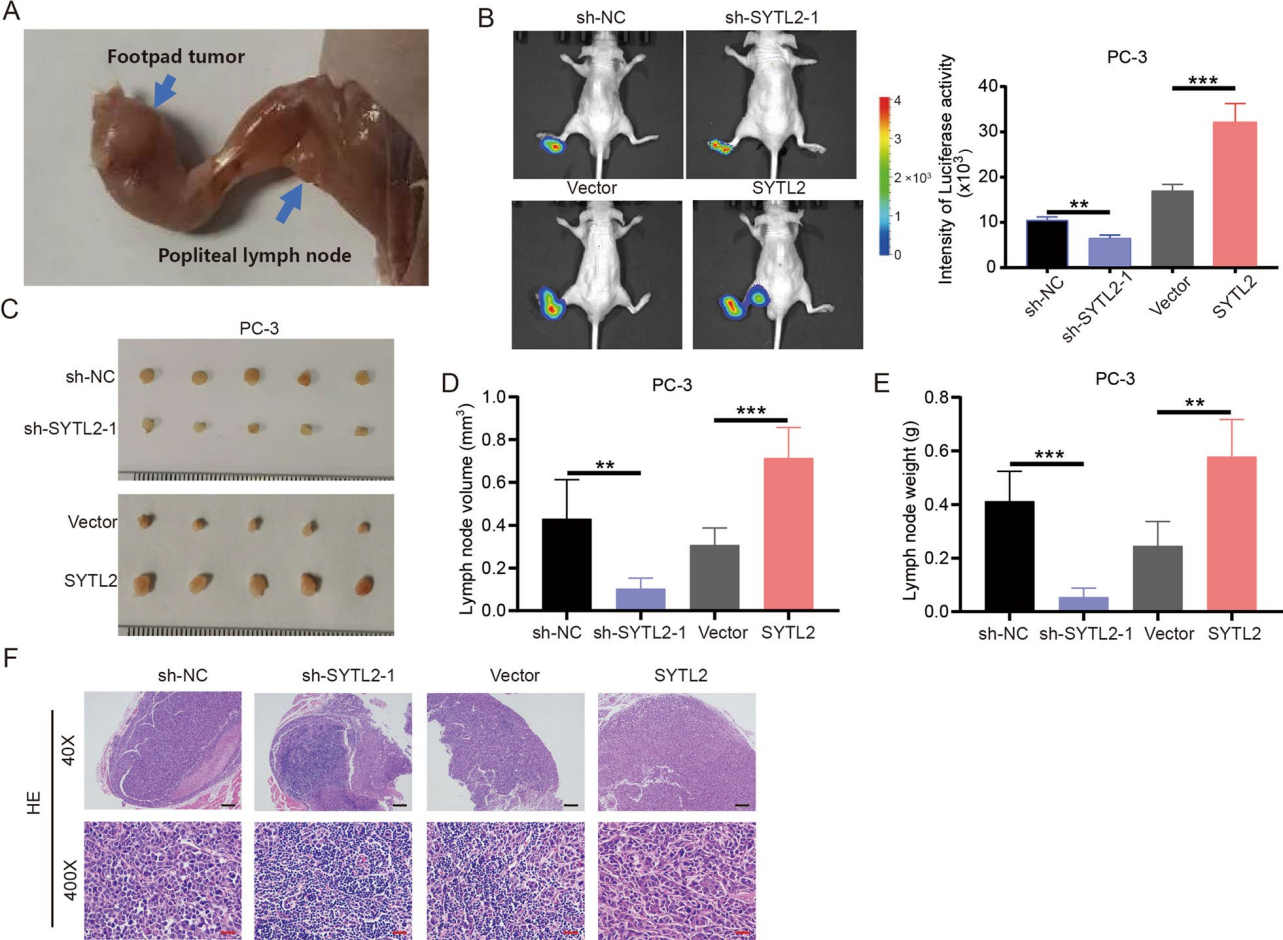
SYTL2 facilitates lymph node metastasis of PCa cells in vivo*.* **A** Representative images of the nude BALB/c mouse model of popliteal LN metastasis. The indicated PC-3 cells were injected into the footpads of the nude mice, and the popliteal LNs were enucleated and analyzed. **B** Representative images of bioluminescence in popliteal LN metastasis in the indicated cell groups (n = 5 per group). **C**–**E** Representative images of dissected popliteal LNs (**C**) and quantification analysis of the LN volume (**D**) and LN weight (**E**). **F** Representative images of H&E staining confirming the LN status (n = 5). The black arrow shows the PCa cell. *p < 0.05, **p < 0.01 and ***p < 0.001. Scale bars: 50 μm (red), 500 μm (black)

To investigate the functions of SYTL2 in PCa metastasis in vivo, we constructed a model of popliteal lymph node (LN) metastasis in nude mice with PC3/luciferase (PC3/luc) PCa cell lines (Fig. 3A). We inoculated PC3/luc cell lines with stable knockdown or overexpression of SYTL2 into the footpads of BALB/c nude mice. Eight weeks after inoculation, the status of lymph node metastasis was determined by a bioluminescence imaging system. SYTL2 silencing significantly impeded PC3/luc cell metastasis to LNs. In contrast, a high level of SYTL2 promoted LN metastasis (Fig. 3B). Moreover, the popliteal LNs were dissected for analysis, and the volumes of the popliteal LNs were the largest in SYTL2-overexpressing mice and the smallest in SYTL2 shRNA mice (Fig. 3C–E). H&E staining was performed to confirm the status of LN metastasis (Fig. 3F).

### SYTL2 interacts with FSCN1 and regulates its stability

**Fig. 4:**
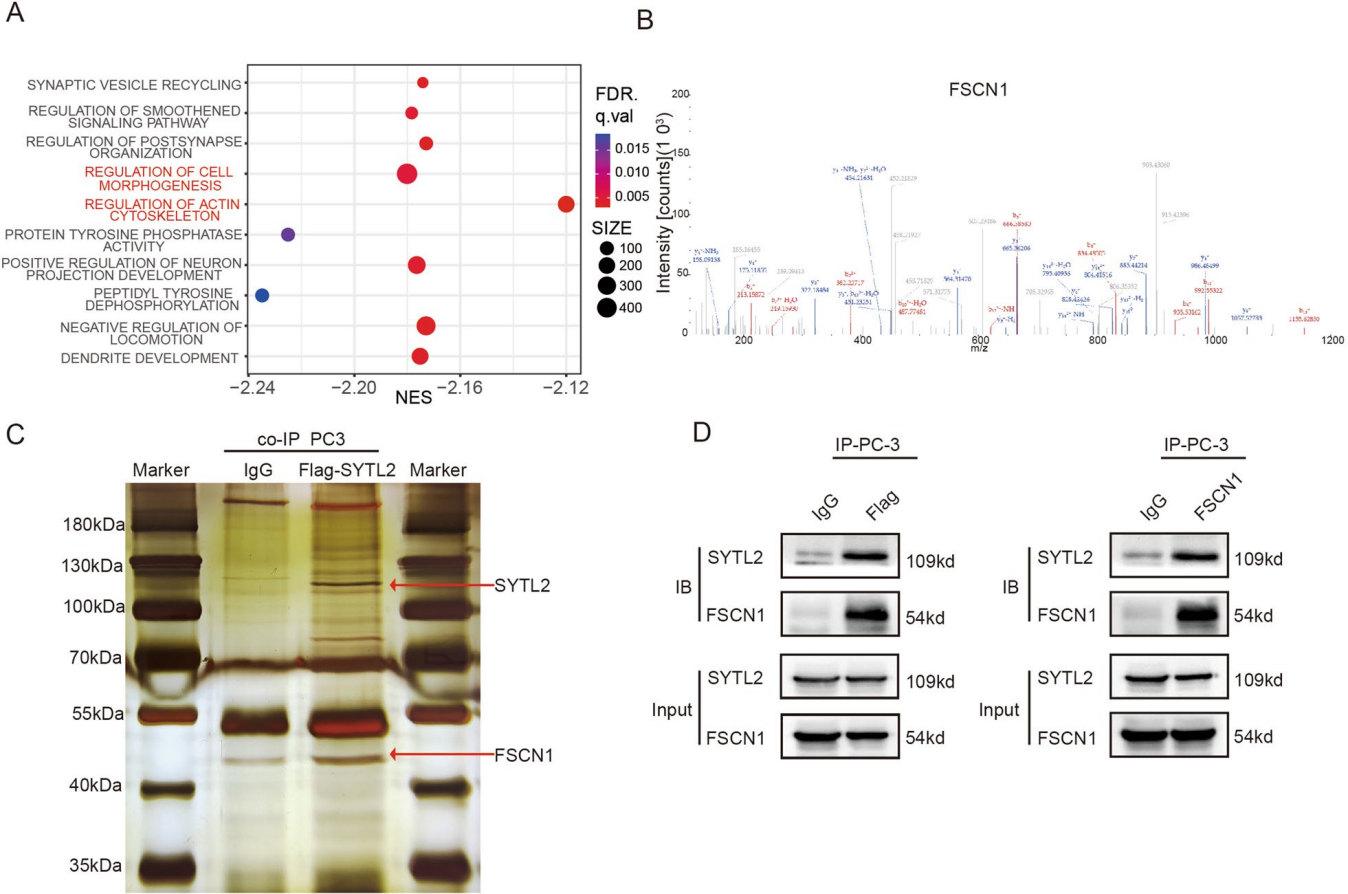
SYTL2 regulates the protein level of FSCN1 by interacting with it. **A** The PCa transcriptome sequence data of TCGA were divided into two groups based on the median expression level of SYTL2 for the bubble plot of nonredundant enrichment clusters of KEGG using GSEA. **B** Representative image of silver-stained SDS‒PAGE gels showing separated proteins that were pulled down using Flag-labeled SYTL2. Anti-IgG was used as the negative control. **C** The mass spectrum of a representative peptide fragment of FSCN1. **D** Western blot analysis determined that SYTL2 interacts with FSCN1 after performing the pull-down assay with Flag-labeled SYTL2 (left panel) and an anti-FSCN1 (right panel) immunoprecipitation antibody. Anti-IgG was used as the negative control protein in the pull-down assay

To further determine the function of SYTL2 in regulating PCa metastasis, the transcriptome data from TCGA were divided into two groups according to the expression level of SYTL2, and the differentially expressed genes were acquired for enrichment analysis. The expression level of SYTL2 was positively correlated with cell morphogenesis and the actin cytoskeleton (Fig. 4A). Meanwhile, a coimmunoprecipitation (co-IP) experiment was performed by using Flag-labeled SYTL2 to identify the proteins that interact with SYTL2 in the PC3 cell line. The magnetic beads were collected for analysis by MS, and an obvious band with a molecular weight between 40 and 55 kDa was observed (Fig. 4B, C).

The proteins detected by MS were screened by their biological function. We found that FSCN1, which is related to actin filament bundle organization, was detected, and the mass spectrum of FSCN1 is shown in Fig. 4C. To confirm the interaction between SYTL2 and FSCN1, the samples from the co-IP experiment using Flag-labeled SYTL2 as bait were detected by FSCN1 antibody, which showed that SYTL2 could interact with FSCN1 and vice versa (Fig. 4D). These results show that SYTL2 can bind to FSCN1.

**Fig. 5:**
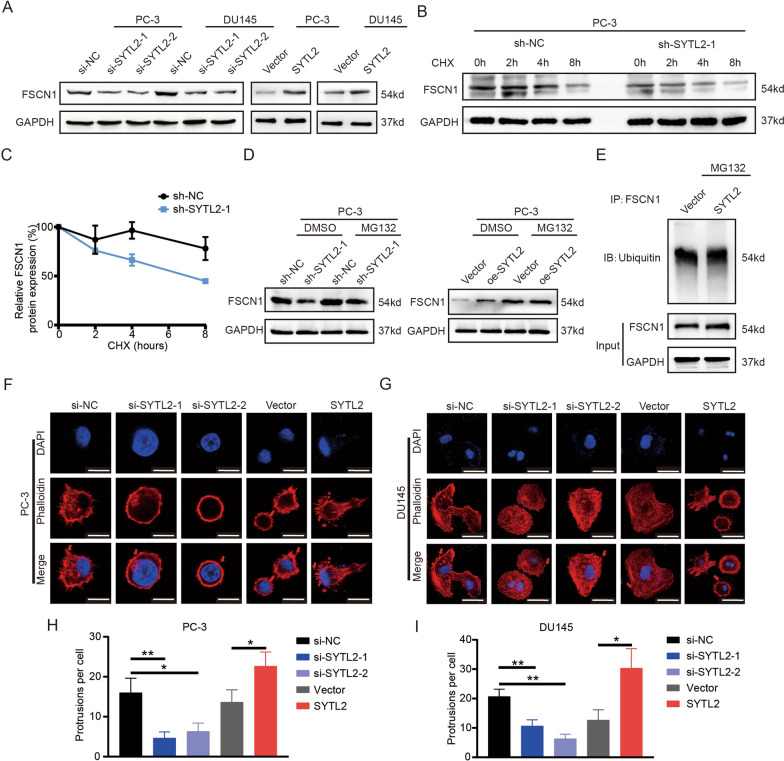
SYTL2 silencing impeded pseudopodia formation and promoted the proteasomal degradation of FSCN1 in a ubiquitin-independent manner. **A** Representative image of the Western blotting analysis of FSCN1 protein levels after SYTL2 knockdown or overexpression in DU145 and PC-3 cells. **B** PC-3 cells with or without SYTL2 knockdown were treated with cycloheximide (CHX) and harvested at different time points. The protein level of FSCN1 was analyzed by Western blotting analysis. **C** The relative protein expression level of FSCN1 was quantitatively analyzed by ImageJ and presented in the degradation curve. **D** PC-3 cells were treated with DMSO (control) or 10 μM MG132 for 6 h. MG132 impeded the alteration in FSCN1 protein expression levels mediated by SYTL2 knockdown (left panel) or SYTL2 overexpression (right panel). **E** To examine the alteration in the ubiquitination level of FSCN1 after changing SYTL2 expression, PC-3 cells transfected with vector or SYTL2 were treated with MG132 (10 μM). The cell lysates were immunoprecipitated with anti-FSCN1 antibodies, followed by immunoblotting using anti-ubiquitin. **F**–**I** Representative image of immunofluorescence in PC-3 (**F**) and DU145 (**G**) cells using the indicated reagents (DAPI shown in blue and phalloidin (shown in red arrow). The protrusions of cells, which represent the ability of pseudopodia formation, were quantitatively analyzed (**H**, **I**). Scale bars: 10 μm (white), *p < 0.05

To investigate the relationship between SYTL2 and FSCN1, cell lysates from PCa cell lines with SYTL2 knockdown or overexpression were detected by WB experiments. We found that overexpressing SYTL2 increased the protein level of FSCN1, while knockdown of SYTL2 reduced the level of FSCN1 (Fig. 5A). To determine whether SYTL2 influences the degradation of FSCN1, we performed a protein stability assay with cycloheximide (CHX). The cell lysates were harvested at different time points after CHX treatment, followed by WB detection. The results are shown in Fig. 5B, which indicates that the degradation rate of FSCN1 accelerated after silencing SYTL2. We next found that in the presence of MG132, FSCN1 degradation was inhibited in SYTL2-knockdown PC3 cells (Fig. 5C). To further investigate how SYTL2 inhibits FSCN1 degradation, we examined the level of FSCN1 ubiquitination regulated by SYTL2 (Fig. 5D). The results revealed that the ubiquitination level of FSCN1 was not altered after SYTL2 overexpression (Fig. 5E). Hence, SYTL2 inhibited the proteasomal degradation of FSCN1 via distinct mechanisms not involving ubiquitination.

### SYTL2 promotes pseudopodia formation and facilitates PCa metastasis in an FSCN1-dependent manner.

Previous studies have indicated that SYTL2 is a regulator of vesicular trafficking involved in cytoskeleton reconstruction [19, 21, 22], and FSCN1 is also an essential factor in the formation of membrane ruffles [15, 16, 34]. We speculated that SYTL2 facilitates PCa metastasis by regulating cytoskeleton reconstruction and promoting the formation of pseudopodia. To test this hypothesis, rhodamine phalloidin was used to indicate the formation of pseudopodia. The IF results revealed that SYTL2 ablation hindered pseudopodia formation, while SYLT2 overexpression increased the number of pseudopodia (Fig. 5F, G).

**Fig. 6:**
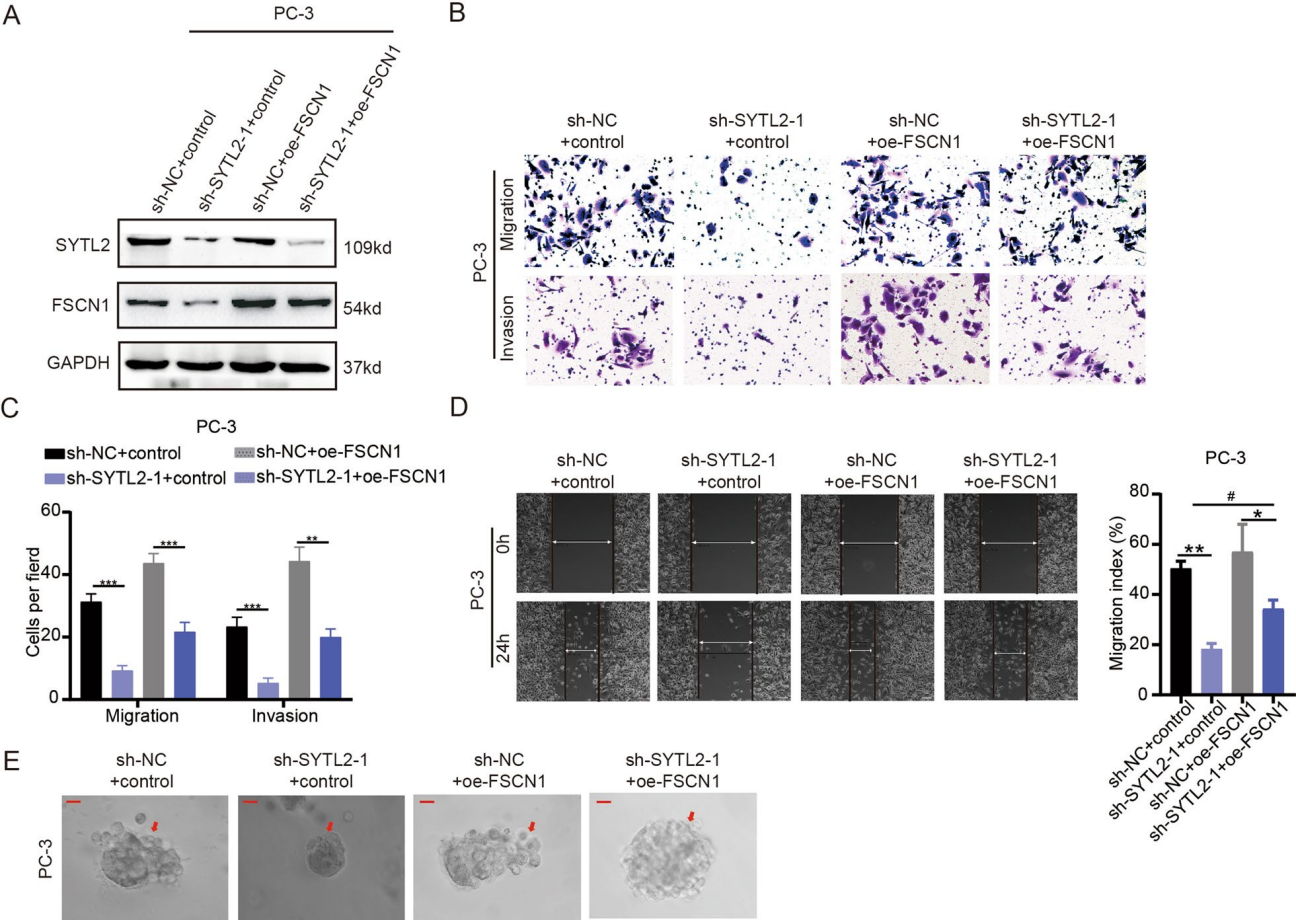
Overexpression of FSCN1 rescues the function in SYTL2-knockdown cells. **A** The levels of SYTL2 and FSCN1 protein were detected by Western blotting in SYTL2-knockdown cells transfected with FSCN1. **B**, **C** Representative images of cell migration and invasion (**B**) were analyzed using SYTL2-knockdown or control cells combined with FSCN1 transfection, and a quantification analysis of the migrated or invaded cell counts (**C**) is shown. **D** Representative images of wound-healing assays using SYTL2-knockdown or control cells combined with FSCN1 transfected in PC-3 cells, showing rescuing cell motility after using FSCN1 plasmid in SYTL2-knockdown cells (left panel) and a quantification analysis of cell migration index is shown (right panel). **E** Representative images of three-dimensional (3D) cell culture using SYTL2-knockdown or control cells combined with FSCN1 transfection showing rescued cell motility after using FSCN1 plasmid in SYTL2-knockdown cells; *p < 0.05, **p < 0.01 and ***p < 0.001. Two-way ANOVA was performed to analyze factorial designed data, ^#^p < 0.05. Scale bars: 10 μm (red)

**Fig. 7:**
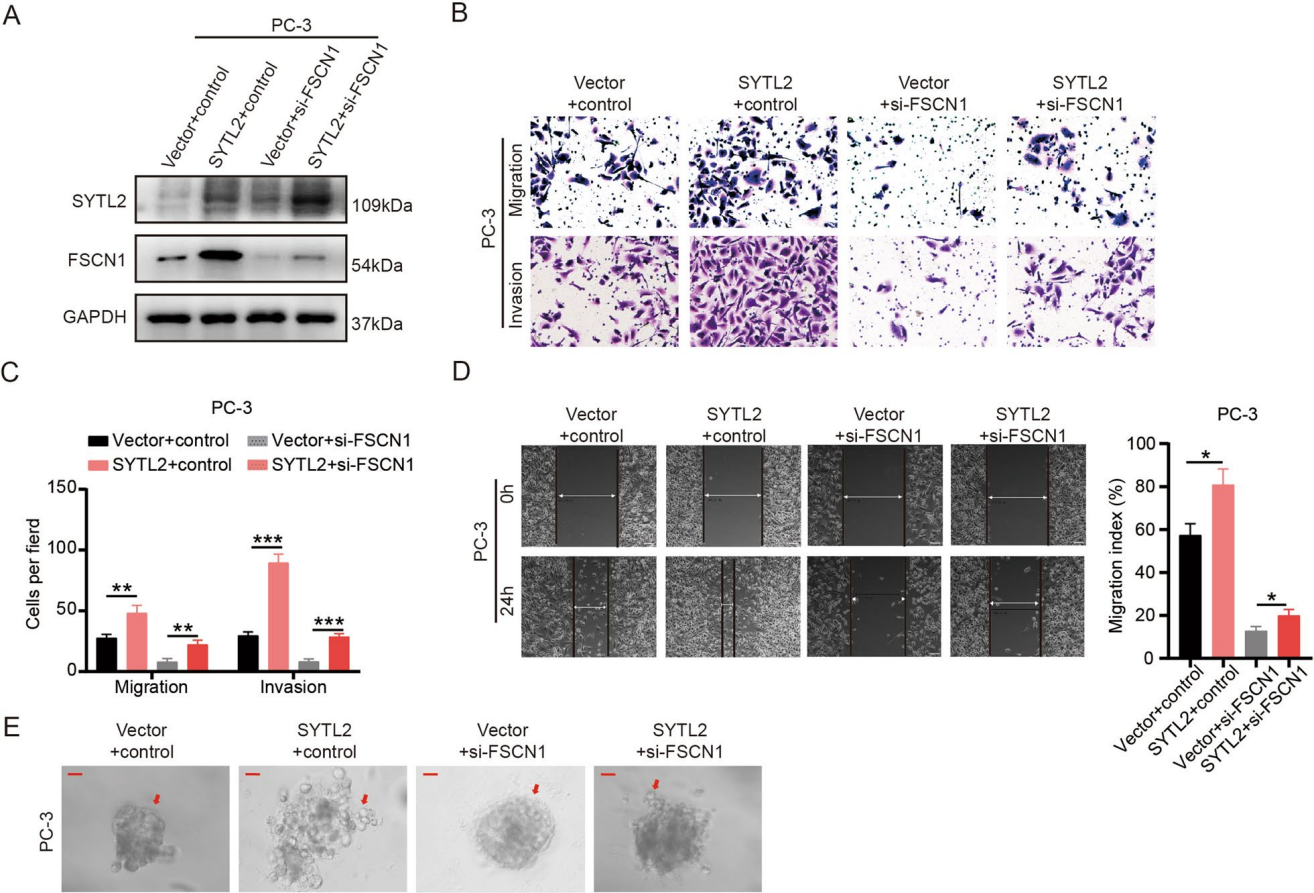
Inhibitor of FSCN1 reverses the function in SYTL2-overexpressing cells. **A** The levels of SYTL2 and FSCN1 protein were detected by Western blotting in SYTL2-overexpressing cells combined with FSCN1 siRNA. **B**, **C** Representative images of cell migration and invasion (**B**) were analyzed using SYTL2-overexpressing or control cells transfected with FSCN1 siRNA, and a quantification analysis of the migrated or invaded cell counts (**C**) is shown. **D** Representative images of wound-healing assays using SYTL2-overexpressing or control cells combined with FSCN1 siRNA transfected in PC-3 cells, showing reversing cell motility after using FSCN1 siRNA in SYTL2-overexpressing cells (left panel) and a quantification analysis of the cell migration index is shown (right panel). **E** Representative images of three-dimensional (3D) cell culture using SYTL2-overexpressing or control cells combined with FSCN1 siRNA transfection showing reversed cell motility after using FSCN1 siRNA in SYTL2-overexpressing cells; *p < 0.05, **p < 0.01 and ***p < 0.001. Scale bars: 10 μm (red)

Moreover, to study whether SYTL2 regulates the metastasis of PCa in an FSCN1-dependent manner, we overexpressed FSCN1 in stable SYTL2-knockdown cells (Fig. 6A). Then, we found that exogenous FSCN1 overexpression rescued the SYTL2 knockdown-induced inhibition of metastasis in PCa cells (Fig. 6B–E). To further confirm the mechanism, FSCN1-specific siRNA was transfected into the SYTL2-overexpressing PCa cell line (Fig. 7A). As expected, silencing FSCN1 attenuated the SYTL2-mediated enhanced metastasis of PCa in vitro (Fig. 7B–E). All of these findings demonstrated that SYTL2 promoted PCa metastasis in an FSCN1-dependent manner.

## Discussion

Cancer metastasis, regulated by tumor-intrinsic and tumor-extrinsic factors, contributes to a higher mortality rate among patients bearing tumors [4–6]. Understanding the mechanism of mPCa is essential for individualized cancer therapy. In the present study, we revealed that a high expression level of SYTL2 was correlated with a high rate of metastasis, an advanced clinical tumor stage and a poor prognosis in PCa samples. Our study also demonstrated that SYTL2 promotes pseudopodia formation in an FSCN1-dependent manner, leading to PCa metastasis.

Metastasis is a complex process in which multiple mechanisms contribute to completion of cancer metastasis. Tumor-intrinsic mechanisms, including oncogenic mutation and epigenetic modification, result in remodeling of the morphology of tumor cells and provide them with mobility and extracellular matrix breakdown abilities. Moreover, tumor-intrinsic regulators also maintain the survival of circulating tumor cells and provide them with the ability to settle in distant organs. At the same time, tumor-extrinsic mechanisms, including tumor-stromal interactions and premetastatic niche formation, enhance cellular differentiation and guide the migration direction [35].

Among these mechanisms, cytoskeleton remodeling and pseudopodia formation are the central steps of morphological changes and the first step of cancer metastasis. Pseudopodia are actin‐rich protrusions that drive cell movement [34, 36, 37]. Several pathways and molecules have been proven to be involved in the cytoskeletal regulation of metastasis by previous studies. The Rho/ROCK signaling pathway is one of the most important pathways in cytoskeleton reorganization [38]. Zheng et al. also noted that silencing N-myc downstream regulated gene-1 (NDRG1) impeded the binding between RhoGDIα and CDC42 to activate the CDC42-PAK1/cofilin signaling pathway and promote the formation of pseudopodia [39]. In the present study, SYTL2 enhanced PCa migration and promoted pseudopodia formation by interacting with FSCN1, which is a member of the fascin family of actin-binding proteins [34, 40]. We determined that SYTL2 serves as a regulator of pseudopodia formation in cancer metastasis.

To further investigate the relationship between SYTL2 and FSCN1, a protein stability assay was performed. Our study revealed that SYTL2 enhances the formation of pseudopodia by impeding the proteasomal degradation of FSCN1. Although we determined that SYTL2 positively regulated FSCN1 by decreasing its proteasomal degradation in a ubiquitin-independent manner, the specific mechanism needs to be elucidated in future research. SYTL2 is a protein containing two major domains, the C2 domain and SLP domain, which contribute to regulating cellular morphology [18, 19]. The morphology of kidney epithelial cells is also regulated by the interaction between the C2 domain and Rap1Gap2 [41, 42]. The SLP domain has a relevant function in combination with the C2 domain [43]. We hypothesize that the SLP domain contained in SYTL2 is a protein‒protein binding domain that interacts with FSCN1 [43].

In addition, we revealed that SYTL2 is an enhancer of PCa metastasis that could be a biomarker for mPCa genotyping and a therapeutic target for mPCa. Moreover, we also found that SYTL2 enhances the mobility of PCa by interacting with FSCN1 and regulating pseudopodia formation. Therefore, small agents targeting cytoskeletal reorganization also have potential as therapeutic drugs for SYTL2-overexpressing mPCa patients.

## Conclusion

**Fig. 8:**
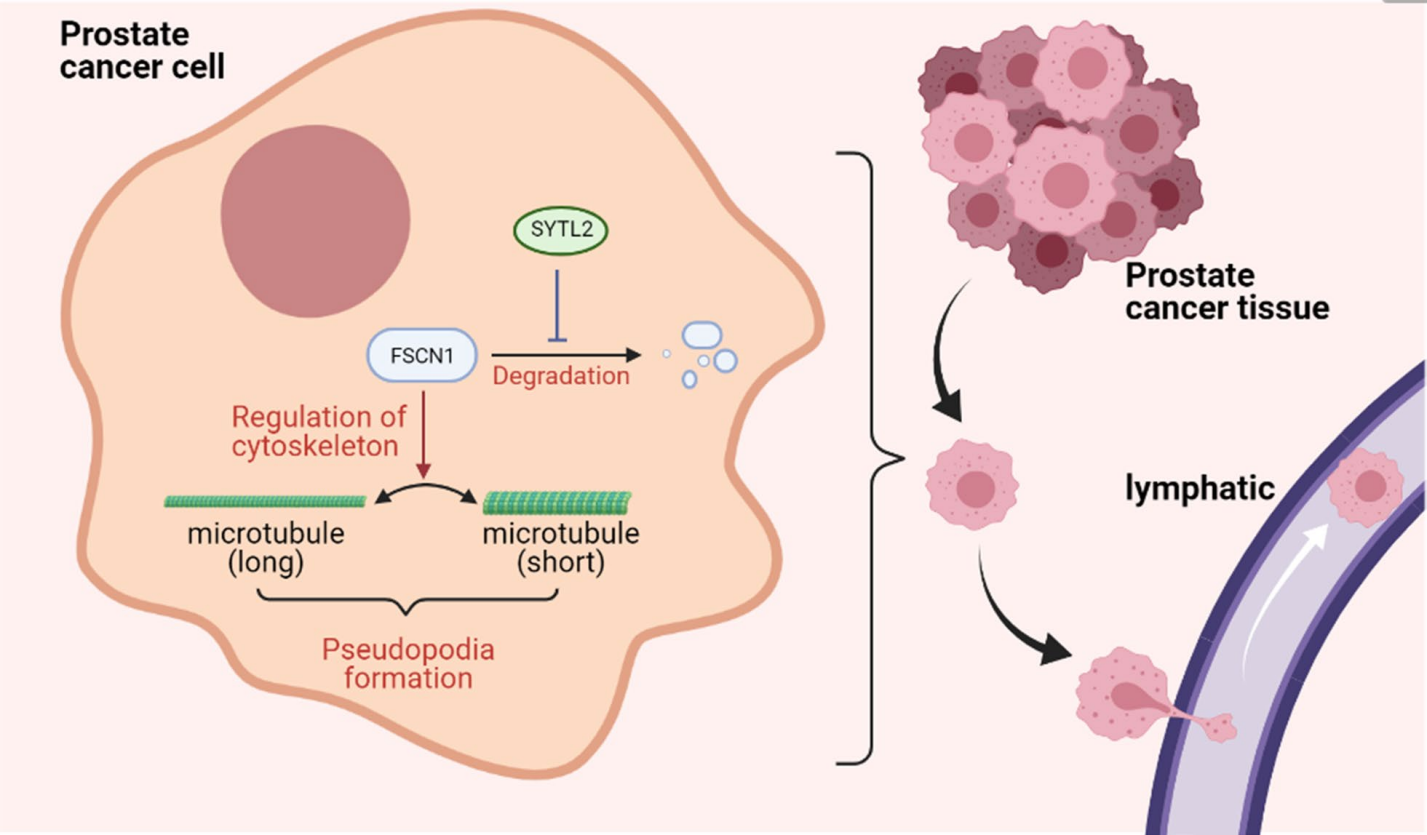
A schematic model of the mechanism underlying the role of SYTL2 in PCa metastasis

We discovered that SYTL2 promotes the formation of pseudopodia and enhances LN metastasis in a novel, Rab27A-independent manner. SYTL2 interacted with FSCN1 and inhibited the proteasomal degradation of FSCN1 via a ubiquitin-independent pathway (Fig. 8). SYTL2 and cytoskeletal remodeling could be therapeutic targets for SYTL2-overexpressing mPCa patients. Therefore, our findings regarding SYTL2 provide a novel approach to the individual treatment of PCa.

## Supplementary Information

**Additional file 1**: **Table S1** The primer, RNAi and shRNA sequences used in this article. **Table S2** The intersection of differential genes among CPGEA, GSE45016 and GSE67872. **Table S3** Associations between SYTL2 expression and clinicopathological characteristics of PCa patients in TCGA database. **Table S4** Associations between SYTL2 expression and clinicopathological characteristics of PCa patients in CPGEA database.

## Data Availability

The transcriptome sequencing data of Chinese patients analyzed during the current study are available in the Chinese Prostate Cancer Genome and Epigenome Atlas (CPGEA): http://www.cpgea.com/. Two datasets (GSE45016 and GSE67872) analyzed during the current study are available in the Gene Expression Omnibus, https://www.ncbi.nlm.nih.gov/geo/.

## Abbreviations

BCR-free survival: Biochemical recurrence-free survival
ATCC: American Type Culture Collection
CHX: Cycloheximide
co-IP: Coimmunoprecipitation
CPGEA: Chinese Prostate Cancer Genome and Epigenome Atlas
Daam1: Dishevelled associated activator of morphogenesis 1
DFS: Disease-free survival
FSCN1: Fascin actin-bundling protein 1
H&E: Hematoxylin–eosin
LN: Lymph node
(m)PCa: (Metastatic) prostate cancer
OS: Overall survival
PPI: Protein‒protein interaction
qRT‒PCR: Quantitative reverse transcriptase-polymerase chain reaction;
TURP: Transurethral resection prostate
siRNA: RNA interference
SLP domain: Synaptotagmin-like protein domain
SPF: Specific pathogen-free
SYTL2: Synaptotagmin-like 2
