# Supplementary material for: SYTL2 promotes metastasis of prostate cancer cells by enhancing FSCN1-mediated pseudopodia formation and invasion
Source: J Transl Med. 2023 May 5;21:303. doi: 10.1186/s12967-023-04146-y (PMC10161564; doi:10.1186/s12967-023-04146-y)
Supplement: Supplementary file 1 — Additional file 1: Table S1 The primer, RNAi and shRNA sequences used in this article. Table S2 The intersection of differential genes among CPGEA, GSE45016 and GSE67872. Table S3 Associations between SYTL2 expression and clinicopathological characteristics of PCa patients in TCGA database. Table S4 Associations between SYTL2 expression and clinicopathological characteristics of PCa patients in CPGEA database. [file 12967_2023_4146_MOESM1_ESM.docx]

**Table S1 The primer, RNAi and shRNA sequences used in this article.**

| **Primer** |  |  |  | |
| --- | --- | --- | --- | --- |
| SYTL2 | Forward | GGAGAGTGAAGGCTCGCAAC | | |
|  | Reverse | TCGCCGTCTCTCTTGTCTTC | | |
| FSCN1 | Forward | CAGCGGCCTCTCGTCTAC | | |
|  | Reverse | GTACTTGTTGCCGCAGTTGA | | |
| GAPDH | Forward | CAAGGCTGAGAACGGGAA | | |
|  | Reverse | TGAAGACGCCAGTGGACTC | | |
|  |  |  |  | |
| **siRNA** |  | **Sequence 5'-3'** |  | |
| Si-NC | | UUCUCCGAACGUGUCACGUTT | |  |
| Si-SYTL2-1 | | CCUUGAAUCCUGUGUAUAATT | |  |
| Si-SYTL2-2 | | GCCUGAAGAUCUGAUGGAATT | |  |
| Si-FSCN1-1 | | CCGACUGCCGUUUCCUCAUTT | |  |
| Si-FSCN1-2 | | GCUGCUACUUUGACAUCGATT | |  |
|  |  |  |  | |
| **shRNA** |  | **Sequence 5'-3'** |  | |
| Sh-NC | | UUCUCCGAACGUGUCACGUTT | |  |
| Sh-SYTL2-1 | | CCUUGAAUCCUGUGUAUAATT | |  |

**Table S2 The intersection of differential genes among CPGEA, GSE45016 and GSE67872.**

| Gene name | GSE67872(log FC,Metastatic vs Normal) | GSE45016 (log FC, Metastatic vs localized) | CPGEA(log FC, Tumor vs Normal) | DSF in TCGA (p value (HR)) | BCRFS in TCGA (p value(HR)) |
| --- | --- | --- | --- | --- | --- |
| SYTL2 | 10.773315 | 1.20662195 | 1.260948771 | 0.0409 (1.554) | 0.0413 (1.832) |
| PCSK6 | 4.528403 | 1.73139997 | 1.407197116 | NS | NA |
| ALCAM | 4.452223 | 1.25104926 | 1.901698929 | NS | NA |
| PPP1R9A | 3.86756 | 1.42507201 | 1.528330317 | NS | NS |
| SAMD5 | 3.805996 | 2.18666743 | 1.772775153 | NS | NA |
| ELF5 | 3.618278 | 1.25754525 | 2.294874757 | NS | NA |
| ASPM | 3.378947 | 1.21041854 | 1.769137685 | NS | NS |
| SRCIN1 | 2.477575 | 1.57286556 | 1.217268737 | NS | NS |
| FOXD1 | 2.27842 | 2.68657057 | 2.739664471 | NS | NA |
| GOLM1 | 2.162821 | 1.37459515 | 1.898513642 | NS | NS |
| ATP8A1 | 2.104765 | 1.47167016 | 1.397343882 | NS | NA |
| GALNT3 | 1.843453 | 1.35936322 | 1.778726363 | NS | NA |
| SLC7A11 | 1.629315 | 1.31816124 | 1.985234481 | NS | NA |
| SNX22 | 1.388958 | 1.27381552 | 1.604244445 | NS | NA |
| CADM1 | 1.321411 | 3.56840561 | 1.28676554 | NS | NA |

DSF: disease-free survival; BCRFS: biochemical recurrence-free survival; HR: Hazard Ratio; NS: no significant; NA: not analysis.

**Table S3 Associations between SYTL2 expression and clinicopathological characteristics of PCa patients in TCGA database.**

| **Clinical feature** | **Total patients n.** | **Low n. (%)** | **High n. (%)** | **P-value** |
| --- | --- | --- | --- | --- |
| **Age** | | | | |
| ＜65 | 323 | 171 | 152 | **0.027** |
| ≥65 | 162 | 70 | 92 |  |
| **Gleason score** | | | | |
| ≤6 | 45 | 31 | 14 | **0.013** |
| 3+4 | 145 | 77 | 68 |  |
| 4+3 | 102 | 50 | 52 |  |
| ≥8 | 193 | 83 | 110 |  |
| **T state** | | | | |
| T1-2 | 186 | 103 | 83 | **0.037** |
| T3-4 | 292 | 136 | 156 |  |
| **Lymph node metastasis** | | | | |
| N0 | 336 | 161 | 175 | 0.397 |
| N1 | 77 | 35 | 42 |  |
| **Distant metastasis** | | | | |
| M0 | 444 | 219 | 225 | 0.512 |
| M1 | 3 | 1 | 2 |  |

A total of 485 samples in TCGA had the expression data of SYTL2. Some samples missed the clinicopathological information in which seven samples lack the T stage information, 72 missed lymph node metastasis information and 38 samples missed the distant metastasis information.

**Table S4 Associations between SYTL2 expression and clinicopathological characteristics of PCa patients in CPGEA database.**

| **Clinical feature** | **Total patients n.** | **Low n. (%)** | **High n. (%)** | **P-value** |
| --- | --- | --- | --- | --- |
| **Age** | | | | |
| ＜65 | 41 | 18 | 23 | 0.454 |
| ≥65 | 93 | 49 | 44 |  |
| **Gleason score** | | | | |
| ≤6 | 10 | 7 | 3 | **0.034** |
| 3+4 | 38 | 25 | 13 |  |
| 4+3 | 25 | 12 | 13 |  |
| ≥8 | 60 | 23 | 37 |  |
| **PSA level (ng/ml)** | | | | |
| ＜10 | 27 | 21 | 6 | **0.005** |
| 10-20 | 42 | 18 | 24 |  |
| ＞20 | 65 | 28 | 37 |  |
| **T state** | | | | |
| T1-2 | 66 | 37 | 29 | 0.226 |
| T3-4 | 68 | 30 | 38 |  |
| **Lymph node metastasis** | | | | |
| N0 | 95 | 46 | 49 | 1 |
| N1 | 18 | 9 | 9 |  |
| **Distant metastasis** | | | | |
| M0 | 67 | 37 | 30 | 0.597 |
| M1 | 15 | 7 | 8 |  |

A total of 134 samples in CPGEA had the expression data of SYTL2. Only one sample missed the information of Gleason score and PSA level. However, “pNx” were labeled in 21 samples and “pMx” were labeled in 43 samples. In addition, nine samples missed the information of Distant metastasis.
